# Supplementary material for: Needs and capabilities for improving poultry production and health management in Indonesia
Source: PLoS One. 2024 Aug 22;19(8):e0308379. doi: 10.1371/journal.pone.0308379 (PMC11340977; doi:10.1371/journal.pone.0308379)
Supplement: S1 Appendix — (DOCX) [file pone.0308379.s001.docx]

**Appendix A. Interview protocol (generic version)**

**Purpose:**

The purpose of interviewing the stakeholder is to understand their needs for information and their capabilities in relation to data with regards to production management, disease surveillance, and AMU/AMR.

**Preamble to interview**

- Introduction to project and interview
  - Name of interviewer and go through the Participant Information Sheet (i.e., describe the project, explain what is expected from the participant and how his information will be used). Answer any question the participant might have.
  - Obtain consent for participation, pictures and recording.
  - Thank the interviewee for participating in the interview.

| **Theme** | **Primary question** | ***Examples/Prompts*** | **Secondary questions*** | **Comments** |
| --- | --- | --- | --- | --- |
| **Transversal topics** | Can you please tell us more about your job and your role in the poultry sector? | 1. *Provide feed/drugs to farmers/companies* 2. *Provide technical advice on farm management* 3. *Perform lab diagnosis* |  | **Objectives of this question:**  - Understand the role of the interviewee in the poultry system  - Understand his interactions with the other components of the system |
| **Transversal topics** | Who are the people, associations or institutions who you work with? | *For example:*   1. *Farm advisors* 2. *Farmers* 3. *Traders* 4. *Veterinary services (government or private)* 5. *Community animal health workers* 6. *Others* | - You indicated that you work with <XX>, can you tell me why?   **TO BE ASKED TO ALL STAKEHOLDERS:**   - Can you please provide the contact details of the people you consider your most important collaborators? | The objective of this question is to identify the relationships of the interviewee with other actors of the poultry sector.  To save interview time, it is recommended that follow-up questions are restricted to one or two key collaborators, and, if applicable, explore why veterinary services are not used. |
| **SUMMARY TRANSVERSAL QUESTIONS** | What are the main challenges you find in your job in relation to poultry production? | *For example (multiple options possible):*   1. *managing diseases in the flock* 2. *sourcing healthy chicks* 3. *accessing medicines* 4. *transportation* 5. *government regulations (red tape)* 6. *input costs like feed, medications,* 7. *other* | - You mentioned that <XX> is a challenge, can you tell me why? - How do you manage this challenge now?   **IF DISEASES ARE NOT MENTIONED**   - Are you facing or have you faced any specific challenges related to poultry health or performance?   **IF DISEASES ARE MENTIONED**   - What are the most important poultry diseases or health issues you are facing?   *For example:*   1. *Newcastle disease* 2. *Gumboro disease* 3. *Avian influenza* 4. *Pasteurellosis* 5. *Colibacillosis* | The objective of this question is to capture any major challenges stakeholders face when performing their job and which ones they consider as priorities. |
| **Disease prevention and control** | Do you perform or support any action to improve the health of chickens and prevent diseases **AND/OR** are you involved in the development of policies related to poultry health? | *For example:*   1. *Research on diseases* 2. *Prescribing drugs* 3. *Providing feed or products to improve performance* 4. *Providing advice or technical support to farmers* 5. *Provide vaccines* | **IF INVOLVED IN PREVENTION:**  You mentioned doing <XX>. Do you work in collaboration, or seek technical support or advice to perform this action?  **IF YES:**   - Who do you ask and why?   **IF NO:**   - Why not?   **IF INVOLVED IN POLICY MAKING:**   - Who else is responsible for developing and/or implementing this policy? - How do you ensure that this policy is effectively implemented? | The objective of this question is to understand the role of the interviewee in disease prevention and how they decide on the measures to be taken. |
| **Disease prevention and control** | Were you ever involved in the management of a disease outbreak in chickens? **If yes,** Can you tell me more about it? | *For example,*   1. *Culling* 2. *Support a farmer* 3. *Provide drugs* 4. *Provide advice on management* | You mentioned you did <XX> to manage the issue. Did you work in collaboration, or seek information and/or technical support to perform this action?  **IF YES:**   - Who did you ask, why and what for?   **IF NO:**   - Why not?   **TO BE ASKED TO ALL STAKEHOLDERS:**  **Did you tell anyone about it?**  **IF TOLD SOMEONE**  You mentioned you told <XX>.   - For what reason(s) did you decide to tell <XX>? - What happened after you reported the issue?   **IF DID NOT TELL ANYONE**   - Why did you decide not to report the problem? - Were there any consequences for not reporting? | The objective of this question is to understand the role of the interviewee in the control of poultry diseases, and how they decide on the actions to be taken.  We also want to identify if they are involved in the reporting of disease outbreaks, and if so how they make the decision of reporting the issue or not. |
| **AMU and AMU stewardship** | Please see some pictures of antibiotics and medicines. Do you prescribe, provide or use any of these? | *Will provide a printed document with pictures of antibiotics commonly used in poultry in ID and VN.*  *E.g.: Put some in the feed he sell*  *Provide AM to farmers* |  | The objective of this question is to rapidly verify that the answers given to the following questions concern antibiotics, rather than other products. We are checking the stakeholder’s ability to differentiate antibiotics from other products, rather than identifying the specific antibiotics used. Not too much time should be spent on this question. |
| **AMU and AMU stewardship** | Can you explain to me how you decide to prescribe, sell, use or not use antibiotics or other medicines in poultry? | *Examples/prompts of reasons for AMU (multiple options possible):*   1. *When chickens are sick (to treat disease in those chickens)* 2. *When chickens are sick ( to prevent other chickens in the flock from getting sick)* 3. *Before birds get sick to prevent disease from occurring* 4. *To increase the growth of chickens*   *Examples/prompts for not using AMU (multiple options possible):*   1. *Unable to purchase (due to lack of pharmacy/vet/drug store, or too expensive)* 2. *Do not think these are needed* 3. *Did not work the last time these were used* | **IF ANTIBIOTICS USED/SOLD:**   - What are the main reasons that you use/prescribe/sell antibiotics? - Who do you sell/prescribe antibiotics to? - How do you decide which antibiotics or medicines to use/prescribe? - Do you seek/provide advice from anyone on which antibiotics or medicines to use/prescribe?   - **If yes,** who and why?   - **If no,** how do you (or the person using antibiotics) decide which antibiotics or medicines to use? - Where do you buy antibiotics or medicines from and why? - Do you find it difficult or convenient to buy antibiotics or other medicines? - What do you think of the cost of antibiotics?   **IF ANTIBIOTICS NOT USED/SOLD:**   - Why not? | The objective of this question is to identify what information the stakeholder uses to decide if, how, and what kind of antimicrobials to use in poultry, where they get their information from, and to identify potential needs to be addressed. |
| **AMU and AMU stewardship** | In your opinion, is there anything that should be changed in the way you or other actors from the poultry sector are using antibiotics? |  | **IF WISH TO CHANGE ANTIBIOTIC USE:**   - Which changes and why?  Do you take any actions to achieve this?  - - **If yes**, what are these?  **If no**, why not? What are the barriers?  - Would it be useful to have a tool that provides information on the amount of antibiotics or other medicines that you use/sell/prescribe?   - Why/why not?   **IF DO NOT WISH TO CHANGE ANTIBIOTIC USE:**   - Why not? - Would it be useful to have a tool that provides information on the amount of antibiotics or other medicines that you use or provide?   - Why/why not? | The objective is to identify if stakeholders are motivated to improve AMU stewardship and if there are any barriers to or opportunities for them to do so. If applicable, it also aims to identify if farmers consider it useful to have information on their AMU. |
| **Health information and monitoring** | In general, where do you get information about poultry diseases and their management (including treatment with medicines)? | *For example:*  *People*   1. *Veterinarians* 2. *Gov animal health workers* 3. *Vet shops* 4. *Other farmers* 5. *Relatives* 6. *Own experience* 7. *Feed dealers* 8. *Pharmacists/drug dealers* 9. *Traders/middlemen* 10. *Farmers association*   *Tools*   1. *iSIKHNAS* 2. *InfoLab* 3. *HPAI PDSR* 4. *SIZE 2.0*   *Other*   1. *Media* 2. *Internet* 3. *Television* 4. *Other* | - Do you find it easy to get information? Why/why not? - Among all the sources you indicated, can you tell me which one(s) you prefer and why? - You indicated getting information from <XX>.   - What kind of information do you get from <XX>?   ***Prompts:***   1. *Disease management information* 2. *Information on potential disease outbreaks in the area* 3. *Guidance on treatment when chickens are sick* 4. *Other*    - How do you use this information? | The objective of this question is to identify the main sources of information about poultry health, the type of information they seek and why, and potential barriers to accessing this information. |
| **Health monitoring and information** | Do you record information on production parameters and health in your or others’ animals? | *For example:*   1. *Mortality* 2. *Disease outbreaks or suspicion* 3. *Weight at slaughter* 4. *Feed/drug consumption* 5. *Vaccination* 6. *Environmental conditions* 7. *Other* | **IF YES:**   - Why? How do you use this information? - Which tool(s) or method do you use to record this information?   ***Prompt:*** *Have you heard of, and/or do you use any of the following tools?*   - *Paper record* - *BroilerX* - *CCF Recording* - *Chickin Smart Farm* - *Farm by Agrinis* - *XL Smart Poultry* - Do you share this information with anyone? What for?/Why not?   ***Prompt:*** *Have you heard of, and/or do you use any of the following systems?*   1. *iSIKHNAS* 2. *InfoLab* 3. *HPAI PDSR* 4. *SIZE 2.0* 5. *Farmers association*  - What difficulties do you face in recording or sharing this information?   **IF NO:**   - Why not? What are the main barriers preventing you from doing this? | The objective of this question is to understand which type of information is already being collected, the tool(s) or methods used for recording the information, and how the information is processed and analyzed. |
| **Health monitoring and information** | Do you record information on treatment with antibiotics in your or others’ animals? | *For example:*   1. *Dates of treatments* 2. *Reasons for treatments* 3. *Type of antibiotic/medicine used, etc.* | **IF YES**   - Why? How do you use this information? - How do you record this information? (e.g., paper log, digital App…) - Do you share this information with anyone? What for?/Why not? - What are the main challenges you face in recording or sharing this information?   **IF NO:**   - Why not? What are the main barriers preventing you from doing this? | The objective of this question is to understand if, how and why information about AMU is already being collected and/or shared, and identify potential barriers to recording and/or sharing information about AMU. |

* Secondary questions are more specific and help direct the interviewee to the objectives of the N&C assessment, allowing them to expand on their answer to the primary question.

**Summary questions:**

The team member responsible for drawing the “mind map” showing the respondent’s contact network during the interview will now present it to the farmer. They will review the information that has been collected so far, and amend or complement it if necessary. It will serve as a visual support to help the respondent reflect on the main issues he/she is facing in his/her job, and what he/she thinks could be done to improve his/her current way of operating.

| **SUMMARY TRANSVERSAL QUESTIONS** | Please see the network map we have prepared showing the different interactions and sharing of information, based on what you have told us. Have we missed anyone? |  |  | The map of networks and communications prepared by one of the observers should be presented to the stakeholder at this point.  The objective of this question is to capture any other actors with whom the stakeholder shares information that we may have missed, and to confirm the information in the map prepared by the observer. |
| --- | --- | --- | --- | --- |
| **SUMMARY TRANSVERSAL QUESTIONS** | Do you feel you have all the resources and information to perform your job in regards to diseases and AMU? |  | **IF YES:**   - What resources and information do you find most useful? - What additional information could help you to better prevent or manage diseases?   **IF NO:**   - What prevents you from getting the information you need? - What could help you to better prevent or manage diseases? | The objective of this question is to capture any major resource or information gaps that have not been identified earlier in the interview. |
| **SUMMARY TRANSVERSAL QUESTIONS** | Is there anyone you know who you think would be interested in participating in our study? |  | **IF YES**  Can you please provide us with their contact details? | This question is to give us the opportunity to perform snowball sampling. |

**End of interview**

Ask the interviewee if there is anything else they would like to discuss and if they have any questions e.g., “We have come to the end of the interview. Is there anything else you would like to say?”, “Do you have any questions?”
