# Supplementary material for: Needs and capabilities for improving poultry production and health management in Indonesia
Source: PLoS One. 2024 Aug 22;19(8):e0308379. doi: 10.1371/journal.pone.0308379 (PMC11340977; doi:10.1371/journal.pone.0308379)
Supplement: S2 Appendix — (DOCX) [file pone.0308379.s002.docx]

**Appendix B. Quote table**

| **Sub-theme** | **ID** | **Quote** | **Interviewee type** |
| --- | --- | --- | --- |
| **Theme 1: An increasingly complex and uncertain business environment** | | | |
| Economic vulnerability of poultry producers | a1 | My challenge is, I don't want the population to grow, no. The important thing is that I can still exist, that's all. Because if I am thinking of developing, it seems to be a very hard challenge, while existing seems to be reachable to me. | Independent layer farmer |
|  | a2 | And it was very painful at that time, because once the price dropped, our population was released, the chicken had no price, eggs had no price, but feed was extremely expensive. If we are not strong, many will close, many will collapse. | Independent layer farmer |
|  | a3 | [if you are] mentally strong, you don't have money, you will collapse. Have money, don't have the mentality, you fall too. Because there are many people who have a lot of money but the mentality of "if this continues, how long will it take, I won't dare" in the end, they all get kicked out. | Independent layer farmer |
|  | a4 | Only big integrators, big companies can survive because they have a lot of money. But small farmers, many of them they close, they sell their cages […]. It’s quite challenging to be a farmer in Indonesia. | Feed company business development manager |
| An unstable and unpredictable market | a5 | That's why the breeders now are like a tossed ship. Like the ship in the middle of the sea doesn't know where it's going, its purpose is not clear. We must survive alone, we must fight alone. | Independent layer farmer |
|  | a6 | The spread is uneven, and because it's uneven, many play on it. So it's not fair for the price. You can get Blitar full of eggs, then they all run to Jogja, then all run out in Jogja. | Pharmaceutical company vet and technical service provider |
|  | a7 | We see layer farmers running their businesses individually, without guidance. These layer farmers sell their products individually so traders can easily influence and cause the decline of egg prices. | Farmer association |
| Difficulties in sourcing farm input | a8 | When we talk about controlling the price of feed, it's now back to the availability, because the majority of the raw materials for feed are imported. So, we can't control its availability. Not to mention the ups and downs of the dollar exchange rate, we also don't know. So yes, it's hard to control. | Pharmaceutical company marketing representative |
|  | a9 | The government imports this GPS [grandparent stock] as if they are doing whatever they want. This means that the need is not calculated, how much is needed for DOC [day old chicks], how many eggs are needed, if I have to import that much, there won't be any. | Independent layer farmer |
|  | a10 | even though we are an agrarian country, yes, Indonesia is a large agricultural land, in my opinion it has not been used properly, especially outside Java. [there is] a lot of land that has not been optimal for cultivation, let's say planting corn. So maybe it's also a problem if we rely heavily on imports | University professor |
| Unequal power dynamics | a11 | in the past the competitors were not as hard as they are now. Not as heavy. Now, the competitors are big companies, maybe because the companies have seen the potential in this sector to be very prospective, so they are also working on it. They also have an upstream to downstream system, they control everything. | Independent layer farmer |
|  | a12 | It is impossible for us small farmers to face them. They have DOC [day old chicks], they have feed, they have medicine, they have vaccines, even the last one, they have all the final results. […] My question is, will integrators replace us after we run out? | Independent layer farmer |
|  | a13 | You should just keep going, don't stop. In fact, when someone stops, the giants enter. | Independent layer farmer |
|  | a14 | My question is, will integrators replace us after we run out? So if there were thousands or millions of farmers scattered throughout the archipelago, now there are only 10 or at most 20 large companies. | Independent layer farmer |
| **Theme 2: Limited collaboration among government, academia, and industry stakeholders** | | | |
| Government perceived to have weak interest and involvement in the poultry sector | b1 | the government only wants cheap food prices without understanding the production costs we experience, this is not nice. But there is no protection for us, no protection for us. Now, when the price of feed is expensive like last time, we have to get it from our own pockets. This isn't right. The government wants cheap [food prices], but from our money, not from the government. This is why we are angry, yesterday we were angry. | Independent layer farmer |
|  | b2 | However, our association still needs to improve our relationship with the government because government policies often do not suit our needs. Of course, the government must consider the interests of prominent business people, but this policy is ultimately not in the interests of our farmers. | Farmer association |
|  | b3 | Because our poultry is approaching the industrial scale, it is very advanced. That's why the government can't keep up with that progress. The government can offset businesses that are still traditional, such as dairy farms or beef cattle farms. Meanwhile, if it becomes an industry, farmers do not need the government. They only need precise regulation from the government. So they talk little with the government. | Poultry shop and farm owner |
|  | b4 | We hope the government will be closer to farmers. However, the government does not upgrade knowledge, so it is left behind. They were confused when we asked them to discuss it because the poultry industry was so advanced. | Poultry shop and farm owner |
|  | b5 | the communication with government also not so close, just very rare. Because what will benefit each other. Association will give information, but ask what the government will give back? | Research institution |
|  | b6 | we just never communicate with them. Except when it comes to licensing issues, or issues regarding the NKV [Nomor Kontrol Veteriner = veterinary control number], we really have to deal with them. | Independent layer farmer |
| Information gaps lead to inadequate management of poultry diseases | b7 | Straight from the beginning from 2006 when avian influenza came in, they [sector 1 and 2] were actually not sharing even data with the government, so even if they would have avian influenza they would just dump the chickens in […] the market chain before they all die. But they wouldn’t share the information with government | Non-governmental organization |
|  | b8 | hardly any reports for avian influenza are coming through this iSIKHNAS system. Sometimes none, sometimes there’s - and it’s not very representative - one district which is reporting 10 cases and the neighbouring district which has AI as well won’t actually report any AI over a five or ten years period | Non-governmental organization |
|  | b9 | Because farmers are closer to suppliers, the government is far behind in scientific advances in poultry. For example, when we experience an outbreak, the government has yet to learn, but we have already sent samples abroad. | Poultry shop and farm owner |
|  | b10 | After all, the government is often not ready, especially when facing poultry disease outbreaks. […] If we weren't prepared, we would have collapsed a long time ago for various reasons. For example, when there was a case of AI or Avian Influenza in Indonesia around 2006. At that time, we needed clarification because the government was not ready to deal with the epidemic. | Farmer association |
|  | b11 | The new AI clade happened and then the response from the government is very slow, or there is no response from the government. […] our government lack the ability to response in order to address issues that are very very urgent like this. | Veterinary association |
|  | b12 | So the government at that time [during the first AI outbreak in 2003], they were unwilling to declare that we have been infected by avian influenza. […] The ability of our government to address this issue was very very weak. | Veterinary association |
| Industry actors perceive academic education and research as disconnected from real-life | b13 | Those of us who are practitioners also have much knowledge […] but no one recognizes it because the knowledge we have we got from the field. […] It's like we are studying poultry science, but there has never been a figure in the poultry industry who teaches. | Poultry shop and farm owner |
|  | b14 | Academics are usually born in a community where a system has been formed. They're the smartest people in the community. […] They feel confident and do not need advice from others. […] That's their weakness. They have a good brain but can't accommodate outside advice […] | Poultry shop and farm owner |
|  | b15 | But they talk […] like a scientist. The way to talk to farmers with low education is different. [… ] Farmers know the clinical theory but don't understand laboratory language, which this kind of farmers doesn't understand. […] The seminars they need are about disease management and production management in a language they can easily digest. | Poultry shop and farm owner |
| **Theme 3: Inadequate on-farm management of poultry production and health** | | | |
| Multiple and increasingly complex health challenges | c1 | the priority diseases for the government are only AI and ND. […] H9N2 AI, IB, infectious bronchitis, inclusion body hepatitis, maybe there is no mortality but a drop in production […] but it is not the priority for the government. Because maybe the government just thinks about the mortality, they forget about the economic impact on the farmer. | Veterinary association |
|  | c2 | The farmer may not be afraid of AI. They may be afraid of IB because it is more complicated while AI is something usual for us. | Independent layer farmer |
|  | c3 | Then what is also interesting is that nowadays, many cases in the field are diseases that are sometimes not specific in poultry. It is different from 10 years ago when the disease, for example ND [Newcastle disease], was very visible. Then the AI [avian influenza], it's obvious. Now, it's a mix, so in this case it can't be observed properly. […] In terms of disease, nowadays cases of disease are increasingly complex, they do not stand alone, so the symptoms are not clear and this does require a lab approach | University professor |
|  | c4 | For sure, the disease is more complex and more troubled. Long ago, production drop was a specific case whereas now there are many IB [infectious bronchitis] variants and many diseases that make unclear symptoms because there are two or three viruses. | Independent farmer |
| Lacking or inadequate management of poultry health | c5 | When we talk about small farms, most of them don't use biosecurity, only spray the environment. Unless there's an outbreak, sometimes people aren't allowed to enter and they are more diligent in spraying their surroundings, for the small ones. | Pharmaceutical company technical service officer |
|  | c6 | The dilemma of being a vet but also a salesperson at the same time, we have to diagnose and treat based on the vet knowledge, yet we also have pressure to sell our product so that we have to work on doing treatment using our product even though sometimes we know that the product is not good enough. | Pharmaceutical company vet and technical service officer |
|  | c7 | Now there are more jobs than graduates. This is what I mean as our economy grows. However, the world of education is still lagging, unable to provide output […] it is now difficult for companies to find middle-upper managers | Poultry shop and farm owner |
|  | c8 | our challenges here, it’s like we are doing firefighting actually, so we are firefighters, we have a problem with equipments, we have a problem with human resources, it’s really still far from perfection. | Pharmaceutical industry association |
|  | c9 | The problem is that for animal health, if you look at the budget here in the Ministry, the budget has been going down year after year, livestock production is much more important than animal health, so it’s partly the problem of the government itself | Non-governmental organization |
|  | c10 | you have to be prepared because in Indonesia the government institution is changing. Maybe every year or perhaps suddenly. Like BPPT [Badan Pengkajian dan Penerapan Teknologi, Agency for the Assessment and Application of Technology] […] They have their own budget, how to expand the district and after they join the team, there is no staff, and there is no human resource, there is no budget. | Research institution |
| Resistance to behavioural changes | c11 | However, you know, breeders have their own principles and they maintain that principle. So sometimes it's hard, it turns into a debate that doesn't even come to an end. Because they feel right, it is difficult to change the mindset of farmers. […] And sometimes that's wrong, because they have the principle of "I've been there for decades, what are you new kids doing", it's sometimes difficult for us to go there for education | Independent farmer |
|  | c12 | most of them have been here for a long time and they put forward the old experience, even though knowledge about this bird is constantly growing. Well, it's like the worker doesn't follow the development, he's still stuck in the past. | Pharmaceutical company marketing representative |
| **Theme 4: Insufficient capacity to collect and use poultry health and production data** | | | |
| Capacity to collect and analyze farm data varies significantly among stakeholders | d1 | However, their record-keeping only covers the interests of each farm individually. Why does this happen? First, farmers at lower levels of MSMEs are usually less educated, and most are still in villages, so they need help understanding | Farmer association |
|  | d2 | Farms will make these observations and record them. It is just that because the farms are diverse, the analysis capabilities of each farm also vary. So, we need to improve the farms' internal surveillance system. | Government (Department of Animal Health  Sub-directorate Surveillance) |
|  | d3 | when I was in ADHPI [association of poultry veterinarians], in the association, there are at least 15 poultry diseases that they can report monthly […]. But it’s only circulated in the internal association, not to the government or to other systems. | Research institution |
|  | d4 | everybody has their own data, ASOHI has their data, PINSAR has their data, GPPU has their own data and other associations have their own data. So how do we compile all this data to serve as a data that can be published and used for a good objective, or for a good purpose. | Pharmaceutical industry association |
|  | d5 | Even with the government, we feel that they might not be able to provide valid data. […] The government needs to be encouraged the importance of actually compiling this data so it can actually be used for something. It’s not just asking for data and then stop there, because they don’t have any system to compile them. | Pharmaceutical industry association |
|  | d6 | Yes, and for average field [population], it is a different case. Because it can be made little so that the tax burden is not too big. So, it can't be valid for that data. | Technical service officer |
|  | d7 | Last year, we had a focus group discussion about the populations because we had overpopulation. But the company they just said “yes, yes, it’s not the real data”. Because when they have [a large number of birds], they have to kill the parent stock. | University professor |
| Dissemination of information relies mostly on informal channels | d8 | The fact that the information mainly spreads from TS [technical services] is true because everyday we visit the farm to gather information from the farmer. Over there, we meet TS [technical services] from other companies […] for example, when one farmer knows that the DOC [day old chicks] price will go up, I will inform it to other farmers. | Pharmaceutical company technical service officer from |
|  | d9 | Information spreads through suppliers. […] If there's a big problem, the supplier usually has a stake. For example, if there is a virus, later, the vaccine supplier will announce to Blitar farmers through a seminar, "So we got data that this virus has started to enter Blitar." […] However, between farmers, no one wants to tell each other. | Poultry shop and farm owner |
|  | d10 | in poultry commercial farms, the services do not come from the government, but come from the private technical services. […] So, they know the data of the disease outbreak, not the government | Research institution |
| Barriers to data sharing | d11 | when you asked us whether we have collected data, data analysis from disease cases in poultry, so we have never done that, because it’s not our domain to do so. So for us, if we perform this role, then people would fear that we are trying to disrupt other people’s backyard. | Pharmaceutical industry association |
|  | d12 | And for sharing the data, using application or online application, several institutions have tried that and the most difficult thing is dealing with farmers’ trust, because […] they are usually reluctant to share about the productivity data […] because it’s dealing with the tax | Non-governmental organization |
|  | d13 | Farmers closed themselves because of pride. […] However, between farmers, no one wants to tell each other. | Poultry shop and farm owner |
|  | d14 | for a small farm you can get disease data by providing service, because they don’t have adequate resource for poultry health. […] But from the bigger farms, they have good poultry health management and can be self-sufficient, so why would they have to report? | Non-governmental organization |
|  | d15 | this is not easy to get the data [from sector 3], they don’t want to share any information, especially if there is no benefit for them. […] the key is I think, how to show and to prove they get the benefit, and how to prove this really secure and confidential. | Veterinary association |
| **Theme 5: Leverage points for development** | | | |
| Strengthening intersectoral collaboration | e1 | [some farmers] think "okay this is true, we must support and must join in together". But those who feel "I can stand on my own" do not realise that next week he will have to die. Well this is difficult, that's it. But we're sure we'll get there. | Independent layer farmer |
|  | e2 | I don't know, just work together. That's why today for livestock people to be able to survive I think there needs to be a forum for a kind of cooperation whose point is to be interconnected. | Independent layer farmer |
|  | e3 | the concern today is actually the middle one, you know. After all, the upper class can monopolize the lower class through government subsidies. While for the middle class one, there is nothing. […] So we support each other | Independent layer farmer |
|  | e4 | But when the price is still low, and we have a contract, we can still help the company to get the same profit by performing well. Moreover, if the price is above the contract, the farmers will benefit more. So it's a good thing for partnership under the plasma system, indeed, in the future, the term is that the farmers does not bear the actual loss. | Poultry company head of broiler farm unit |
|  | e5 | In my opinion, the main issue in business is competition. So when farms deal with the government, even though I [Department of Animal Health] am an independent body, if farms really think the government is corrupt, then they also feel the government is not independent […] if you want to use the Public Private Partnership approach, the main problem is information confidentiality and good governance. | Government  (Department of Animal Health  Sub-directorate Surveillance) |
|  | e6 | It would be better if the, the government could support us. For FMD for example, […] if they worked with us, if they collaborated with us, they could work faster because we have the laboratories so we could help them. But I think they don’t trust us yet. | Pharmaceutical company |
|  | e7 | we work closely with the Ministry of Agriculture, the Ministry of Trade, and the Coordinating Ministry for Economic Affairs. We are now also collaborating with universities in mutually beneficial cooperation. We need university assistance for research, understanding poultry health, and farm management. | Farmer association |
|  | e8 | we have a lot of good things in Indonesia but usually we don’t know what is there in the research institutions. We don’t know what the researchers are doing. […] If the government can serve as a bridge, we would be ready to help and to assist. | Pharmaceutical company |
| Addressing education and research gaps | e9 | It is the responsibility of integrators to make sure that their plasma farmers are mentored or are guided properly in the use of product antimicrobial. […] And the more veterinarians who attended our certification for technical trainings, we have a better first line of defence. The hope is that these veterinarians will also provide education for farmers | Pharmaceutical industry association |
|  | e10 | I have an inhouse vet, the inhouse vet, but in my experience, they rarely participate in outside training, you know. So it means that they rarely attend seminars, outside training, so maybe they don't have [scientific] updates. | Independent layer farmer |
|  | e11 | These past few years, I have tried to educate middle to lower-level farmers whose animal health awareness still needs to improve. […] However, my efforts are still very far away. […] The seminars they need are about disease management and production management in a language they can easily digest. | Poultry shop and farm owner |
|  | e12 | what is most important is that Indonesia is actually very rich, there are many natural products that have not actually been looked at. We ask maybe something like MBM, Meat Bone Meal, I ask if it can be replaced with what there is in Indonesia. | Independent layer farmer |
|  | e13 | in Indonesia there is not much research in the field of poultry. Even though we are number four in Asia if I'm not mistaken, we are number eight in the world, yes, it is quite large but research in the poultry sector is not, it is not well developed, right? So it proves that it is not easy to find funding for research in poultry. […] And indeed, that is our weakness, even though the poultry industry in Indonesia is quite large, the research is not yet developed. | University professor |
| Improving data integration and real-time surveillance | e14 | We have recordings every day for our daily productions. The point is that today we are actually told to be really detailed for all the work, if we are not detailed, it's a loss of profit. […] This is the main thing, those who don't want to deal with details will gradually be crushed, it will be difficult to develop. | Independent layer farmer |
|  | e15 | in the past, the key to success was having a lot of information. Now the key to successful breeding is not just a lot of information but must be able to absorb information that is really valid and applied. | Independent layer farmer |
|  | e16 | Like it or not, poultry companies who want to export their products will realize that their farm must become a compartment [for AI] because that is the requirement everywhere. […] Farms are competing with each other, right? If competitors have become AI-free compartments while their farms have not, they will lose their market. | Government (Department of Animal Health,  Sub-directorate of Surveillance) |
|  | e17 | now we only know when the disease is here. But the disease is not just there all of a sudden, there is this process, and we need to know this process […] If we have all the data from the farmer side that would be very helpful. […] what do we need for the future? We don’t want to fight fires, once we have fires and we fight it no we don’t want to do this. We want to know the pattern so that we can be better prepared. | Pharmaceutical company |
|  | e18 | I think that it’s not about data needs, well we always need to know data, but the real-time nature of the data, data needs to be quick to be obtained | Farmer association |
