# Supplementary material for: Needs and capabilities for improving poultry production and health management in Indonesia
Source: PLoS One. 2024 Aug 22;19(8):e0308379. doi: 10.1371/journal.pone.0308379 (PMC11340977; doi:10.1371/journal.pone.0308379)
Supplement: S3 Appendix — (DOCX) [file pone.0308379.s003.docx]

**Appendix C. Codebook**

| **Theme 1: An increasingly complex and unstable business environment** | | |
| --- | --- | --- |
| **Sub-theme** | **Code** | **Ideas to include** |
| Economic vulnerability of poultry producers | Decrease in the number of smallholders | Small farmers going out of business or reducing activity in response to economic stress |
|  | Economic impact of COVID | Farmers going bankrupt, economic losses, reduction of poultry population, staff reduction, disruption of transportation, drop in demand |
|  | Low profit margins | High production costs vs. low egg/meat prices |
|  | Limited financial capacity | Limited cash flow, inadequate financial management skills |
|  | Uncertainty regarding the future | Lack of development prospects, inability to grow, unpredictable future |
| An unstable and unpredictable market | Issues of supply and demand | Overpopulation vs. low demand, uneven geographical distribution of production |
|  | Large fluctuations in market prices | Geographical and seasonal variations, volatility of input and output prices |
|  | Lack of market regulation | No official control over prices and population, fraudulent practices, lack of certification schemes, no official benchmarking |
|  | Hard-to-reach and heterogeneous trade standards | High food safety standards for international trade or high-value markets, differences between regions or countries |
| Difficulties in sourcing farm input | Producers experiencing difficulties in sourcing DOCs | High prices of DOCs, shortages of DOCs, low quality, competition for buying |
|  | Producers experiencing difficulties in sourcing feed | High prices of raw materials, low quality, price instability, insufficient local production, logistical constraints (e.g., transportation), absence of local alternatives |
|  | Inadequate policies | Insufficient import quotas, ineffective subsidies scheme |
|  | High dependence on imports and global markets | Local prices affected by variations of the dollar rate, global shocks (e.g., Russian war) |
| Unequal power dynamics | Smallholders struggle to compete against integrators | Integrators getting bigger and overtaking smallholders, market saturation in some areas |
|  | Integrators have the monopoly over farm input and market prices | Monopoly over feed raw materials, bundling of feed and DOCs or medicine, lobbying, control over market prices |

| **Theme 2: Limited collaboration among government, academia and industry stakeholders** | | |
| --- | --- | --- |
| **Sub-theme** | **Code** | **Ideas to include** |
| Government perceived to have weak interest and involvement in the poultry sector | Limited interactions with poultry stakeholders | Low interest in the poultry sector, priority on cattle, infrequent interaction with stakeholders |
|  | Inadequate understanding and response to industry needs | Government is behind in terms of knowledge, policies and interventions don’t fulfill the needs |
|  | No perceived benefits from interacting with the government | Strong autonomy, perceived disruption to business, defiance, lack of mutual trust |
| Information gaps lead to inadequate management of poultry diseases | Private stakeholders are not open to health authorities | No report to health authorities, incomplete or inaccurate official data on diseases |
|  | The official surveillance system iSIKHNAS is ineffective | Government has insufficient resources for surveillance, low uptake of iSIKHNAS by private stakeholders |
|  | Lack or inadequate management of disease outbreaks | Failure to manage the 2003 HPAI crisis, measures initiated by private sector without legal framework |
| Industry actors perceive academic education and research as disconnected from real-life | Academia does not communicate adequately with industry stakeholders | Inadequate level of language, lack of communication of research output |
|  | Academia is closed to actors from the industry | Reluctance to collaborate with actors outside academia, no participation of industry stakeholders in education |
|  | Academic knowledge is behind and not rooted in real-life | Academia’s knowledge is behind compared to the industry, teaching in universities does not address real-life situations |

| **Theme 3: Inadequate on-farm management of poultry production and health** | | |
| --- | --- | --- |
| **Sub-theme** | **Code** | **Ideas to include** |
| Multiple and increasingly complex health challenges | Highly prevalent or impactful health issues | Health issues perceived as priorities, experience of past outbreaks, impacts of diseases on production |
|  | Environmental risk factors | Exposure to extreme environmental conditions, bad feed and water quality, high farm/poultry densities |
|  | Diseases are becoming increasingly complex and difficult to diagnose | Lack of clear clinical signs due to vaccination or pathogen evolution, no lab diagnosis |
|  | Reduction in disease occurrence or severity | Diseases becoming less frequent due to better biosecurity or vaccination, better experience of managing diseases |
| Lacking or inadequate management of poultry health | Lack of resources for health management | Lack of lab facilities, insufficient financial and human resources |
|  | Farmers rely on their own experience for managing diseases | No lab diagnosis, not seeking vets or technical services, decision based on previous experience |
|  | Inadequate biosecurity practices | Bad sanitation, no use of all in-all-out, mixing of age groups, no pest control |
|  | Low efficacy of vaccines or drugs | Vaccination not wholly effective against new strains, improper prescription and use of drugs due to vested interests |
|  | Insufficient support from health authorities | Limited budget, insufficient staff, frequently changing political environment disturbing public health programs |
| Farmers are resistant to behavioural changes | Lack of technical skills or knowledge | Lack of knowledge about diseases, feed formulation or treatment |
|  | Low awareness or interest in poultry health | Priority given to production, habituation to the risk of diseases, fatalistic attitude, carelessness regarding biosecurity |
|  | Unwillingness to change habits | Old generation vs. millennials, reluctance to follow technical service officers’ recommendations, reliance on own experience |

| **Theme 4: Insufficient capacity to collect and use poultry health and production data** | | |
| --- | --- | --- |
| **Sub-theme** | **Code** | **Ideas to include** |
| Capacity to collect and analyze farm data varies significantly among stakeholders | Low data awareness & literacy | Low awareness or interest in data, lack of analytical skills |
|  | Limited resources for data management | Paper-based recording, lack of digital infrastructure, lack of human and financial resources, sub-optimal data management by the government |
|  | Poor data usability | Low data quality or validity, incompleteness, untimeliness |
| Dissemination of information relies mostly on informal channels | Wide use of informal communication channels | WhatsApp groups of farmers and staff, informal discussions, reliance on social networks |
|  | Central role of technical service officers in information networks | TSO having better access to information, TSO spreading information between stakeholders, report of diseases to TSO |
| Barriers to data sharing | Closed attitude of stakeholders | Health and production data that are considered sensitive, concerns over confidentiality, need to build trust |
|  | Lack of standardization and integration | Discrepancies between private and government data, no official standards, multiple siloed systems |
|  | Negative impact of data-sharing on business | Competitive disadvantage, loss of personal prestige, culling of birds, taxation |
|  | No perceived benefit of sharing data | No need for external support, not knowing how to use the data, authorities giving nothing in return |

| **Theme 5: Leverage points for development** | | |
| --- | --- | --- |
| **Sub-theme** | **Code** | **Ideas to include** |
| Strengthening intersectoral collaboration | Actions to improve communication between public and private stakeholders | Organization of multistakeholder forums or seminars, mediators who facilitate communication, willingness to share information |
|  | Collaboration between public and private actors on research or policy-making | Universities collaborating with large integrators for R&D, farmers participating in research, associations or institutions (e.g., FAO) contributing to policy-making |
|  | Smallholders grouping into partnerships, associations or cooperatives | Creating farmers cooperatives, partnerships with input providers, public-private partnerships, nucleus-plasma system, sustainable financing systems |
|  | Request for government support through better regulation and subsidy schemes | Subsidies, regulation on prices, support research projects, funding |
| Addressing education and research gaps | Associations and integrators provide training and education to farmers | Organization of seminars, technical advice given to farmers |
|  | Knowledge and training gaps in farm and health management | Farmers requesting training on financial management, need to raise awareness on biosecurity, need to focus on smallholders |
|  | Poultry research is insufficiently developed | Need for innovations (e.g., feed additives, alternatives to imported ingredients), insufficient knowledge of new pathogen strains, insufficient budget |
| Improving data integration and real-time surveillance | Stakeholders’ data awareness is increasing | Farmers consider recording as important, willingness to share their data |
|  | Existing sources of data | Existing public and private databases, accessibility of data |
|  | Stakeholders are moving towards digitalized and integrated systems | Use of Excel spreadsheets, tailor-made App for internal use |
|  | Improved data management is driven by business development and regulation | Requirements for compartmentalization, access to markets, access to government subsidies |
|  | Stakeholders are carrying out disease surveillance activities to various extents | Farmers conducting routine titre checks, integrators having dedicated staff, increased access to lab infrastructure, services from input providers or universities |
|  | Potential use of data to support business development | Forecasting & planning of production, evaluation of performance, early warning, provision of better services to customers (e.g., vaccination programs, feed formulation), assessment of drugs or vaccine efficacy, identification of priority issues, benchmarking, disease investigation |
|  | Need for timely health, production, and market data | Need for disease information to improve preparedness, need for price & population data to better manage supply and avoid price fluctuations, need access to others’ data, need real-time data |
